# Case Report: Sequential PTCD and biliary seed stent combined with targeted-immunotherapy for advanced pancreatic cancer with malignant obstructive jaundice: a multidisciplinary approach

**DOI:** 10.3389/fonc.2025.1649080

**Published:** 2025-08-19

**Authors:** Weijun Li, Guoyang Sun, Rui Zhu, Pindong Li, Li Wang

**Affiliations:** ^1^ Department of Emergency Surgery, Union Hospital, Tongji Medical College, Huazhong University of Science and Technology, Wuhan, China; ^2^ Vascular Surgery, The Sixth Hospital of Wuhan, Affiliated Hospital of Jianghan University, Wuhan, China; ^3^ Department of Integrated Traditional Chinese and Western Medicine, Union Hospital, Tongji Medical College, Huazhong University of Science and Technology, Wuhan, China; ^4^ Cancer Center, Union Hospital, Tongji Medical College, Huazhong University of Science and Technology, Wuhan, China

**Keywords:** malignant obstructive jaundice, pancreatic cancer, percutaneous transhepatic cholangial drainage, targeted-immunotherapy, multidisciplinary strategy

## Abstract

Malignant obstructive jaundice (MOJ) due to tumor compression or invasion of the bile duct carries a grave prognosis. We report a case of a 54-year-old female patient (height: 160 cm, weight: 55 kg, BMI: 21.5 kg/m², ECOG performance status: 1, with type 2 diabetes mellitus) advanced pancreatic head cancer causing MOJ, managed with a multidisciplinary approach. Initial endoscopic retrograde cholangiopancreatography (ERCP) with an 8.5 Fr plastic stent failed due to occlusion after 20 days, leading to bilirubin rebound. Emergency percutaneous transhepatic cholangial drainage (PTCD) followed by biliary metal stent (8 mm × 80 mm) and iodine-125 seed implantation effectively relieved obstruction, reducing total bilirubin (TBIL) from 116.9 to 45.6 μmol/L within seven days. Subsequent tomotherapy (TOMO, 66 Gy to gross tumor volume) and a personalized regimen of S1 (tegafur, 20 mg/day), nimotuzumab, and pembrolizumab, following intolerance to gemcitabine + nab-paclitaxel (AG), achieved a 78% reduction in CA19–9 and sustained biliary patency. At one-year follow-up, TBIL was 18.2 μmol/L, direct bilirubin (DBIL) was 9.8 μmol/L, and the patient reported a good quality of life (Karnofsky score: 90). This case demonstrates the efficacy of sequential PTCD, seed stent, and targeted-immunotherapy, offering a practical model for managing advanced pancreatic cancer with MOJ.

## Introduction

Malignant obstructive jaundice (MOJ) results from tumor compression or infiltration of the intrahepatic or extrahepatic bile ducts, most commonly caused by pancreatic head cancer, cholangiocarcinoma, or metastatic disease (1). Its insidious onset means that 60%–80% of patients are diagnosed at an advanced stage, rendering curative surgery unfeasible (2). MOJ leads to bile stasis, which impairs liver function, diminishes cytochrome P450 activity, and restricts the metabolism of chemotherapeutic agents such as oxaliplatin, increasing toxicity risks (3). Elevated bilirubin levels also trigger systemic inflammation, suppress CD8+ T-cell proliferation, and reduce responsiveness to PD-1/PD-L1 inhibitors (4). Furthermore, bile acid deficiency worsens cachexia, while reduced clotting factor synthesis heightens bleeding risks after radiotherapy, limiting the scope of multidisciplinary treatment (5). Rapid relief of biliary obstruction and restoration of liver function are thus pivotal in palliative care (6).

Percutaneous transhepatic cholangial drainage (PTCD), guided by imaging, effectively reduces bilirubin levels, particularly in high-level obstructions or complex hilar tumors (7). However, standalone external or internal-external drainage offers only temporary relief and fails to address tumor progression or restenosis (8). Recent advancements in biliary metal stents combined with iodine-125 seed implantation provide a dual mechanism of mechanical expansion and localized radiotherapy, restoring physiological bile flow, curbing tumor growth, and extending stent patency to 10–12 months (9). Tomotherapy (TOMO) delivers precise radiation to tumor-infiltrated regions and high-risk lymph nodes (10), paving the way for targeted and immunotherapies (11). This report explores the efficacy of PTCD followed by biliary seed stent placement, TOMO, and targeted-immunotherapy in advanced MOJ, providing evidence to support a treatment window for such patients.

## Case presentation

A 54-year-old woman (height: 160 cm, weight: 55 kg, BMI: 21.5 kg/m², ECOG performance status: 1, with type 2 diabetes mellitus, no hypertension) presented with progressive jaundice of the skin and sclera for over one month. Enhanced abdominal CT revealed a 35 mm × 24 mm irregular soft-tissue mass in the pancreatic uncinate process, invading the left renal vein and the space adjacent to the inferior vena cava. Endoscopic ultrasound-guided biopsy confirmed adenocarcinoma, with immunohistochemistry revealing high P53 mutation expression and elevated Ki67 proliferation. Laboratory findings indicated severe MOJ (total bilirubin [TBIL] 218.1 μmol/L, direct bilirubin [DBIL] 122.0 μmol/L), with CA125 at 46.0 U/mL and CA19–9 exceeding 1200 U/mL. The patient had previously undergone endoscopic retrograde cholangiopancreatography (ERCP) at an external hospital, where an 8.5 Fr plastic stent was placed, initially reducing jaundice. However, bilirubin levels rebounded after 20 days, and conservative management failed, prompting transfer to our center.

**Figure 1:**
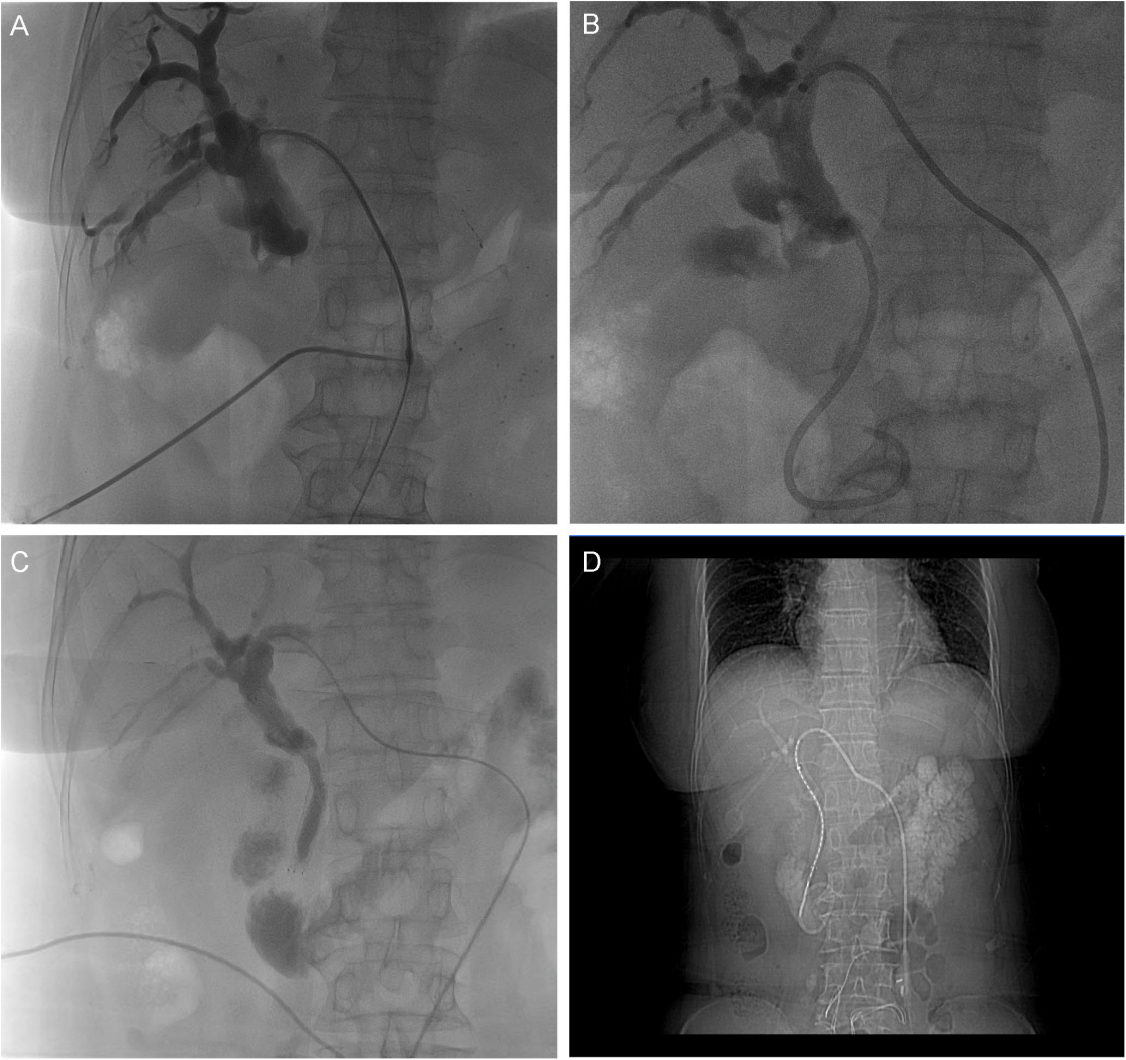
DSA-Guided PTCD and Sequential Stent-Seed Implantation. **(A)** Cholangiography reveals dilated intrahepatic bile ducts and a distal common bile duct cutoff, with no contrast flow to the duodenum. **(B)** Internal-external drainage catheter positioned post-placement. **(C)** 8×80 mm metal stent deployed across the stricture. **(D)** Iodine-125 seed strand implanted along the stenotic segment via PTCD tract.

**Figure 2:**
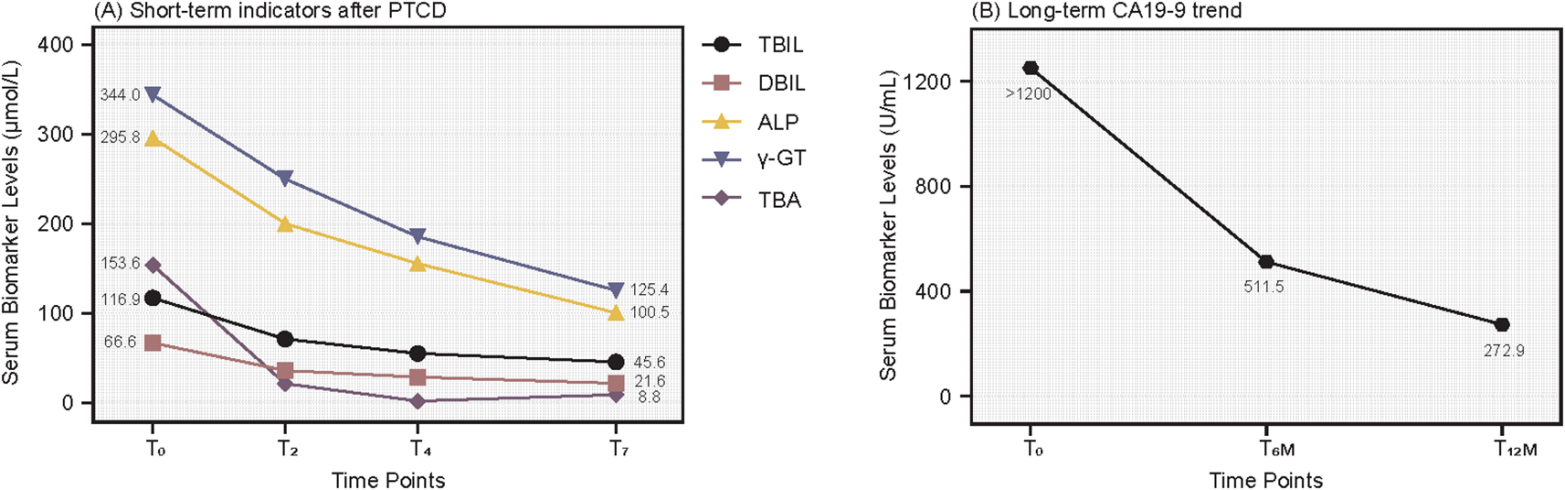
Dynamic Changes in Biliary Obstruction Markers and CA19-9. **(A)** Short-term response: TBIL and DBIL decline rapidly at day 2 (T_2_), day 4 (T_4_), and day 7 (T_7_) post-PTCD, indicating effective decompression. **(B)** Long-term tumor marker response: CA19–9 decreases steadily at 6 months (T_6m_) and 12 months (T_12m_), with remission sustained at one year.

We performed emergency PTCD with internal-external drainage. Under ultrasound guidance, a COOK puncture needle was inserted into a secondary branch of the right hepatic duct, followed by placement of a COOK sheath with digital subtraction angiography (DSA) assistance. Cholangiography showed dilated intrahepatic bile ducts and a distal common bile duct cutoff (**Figure 1A**). A guidewire was navigated through the stenotic segment, and an internal-external drainage tube was placed (**Figure 1B**), yielding approximately 300 mL of daily drainage. Within seven days, TBIL decreased from 116.9 to 45.6 μmol/L (**Figure 2A**). In a subsequent procedure, an 8 mm × 80 mm metal stent was deployed along the guidewire (**Figure 1C**), and a 0.6 mCi iodine-125 seed strand was implanted in the stenotic segment under CT guidance (**Figure 1D**). The occluded plastic stent was removed endoscopically during PTCD.

**Figure 3:**
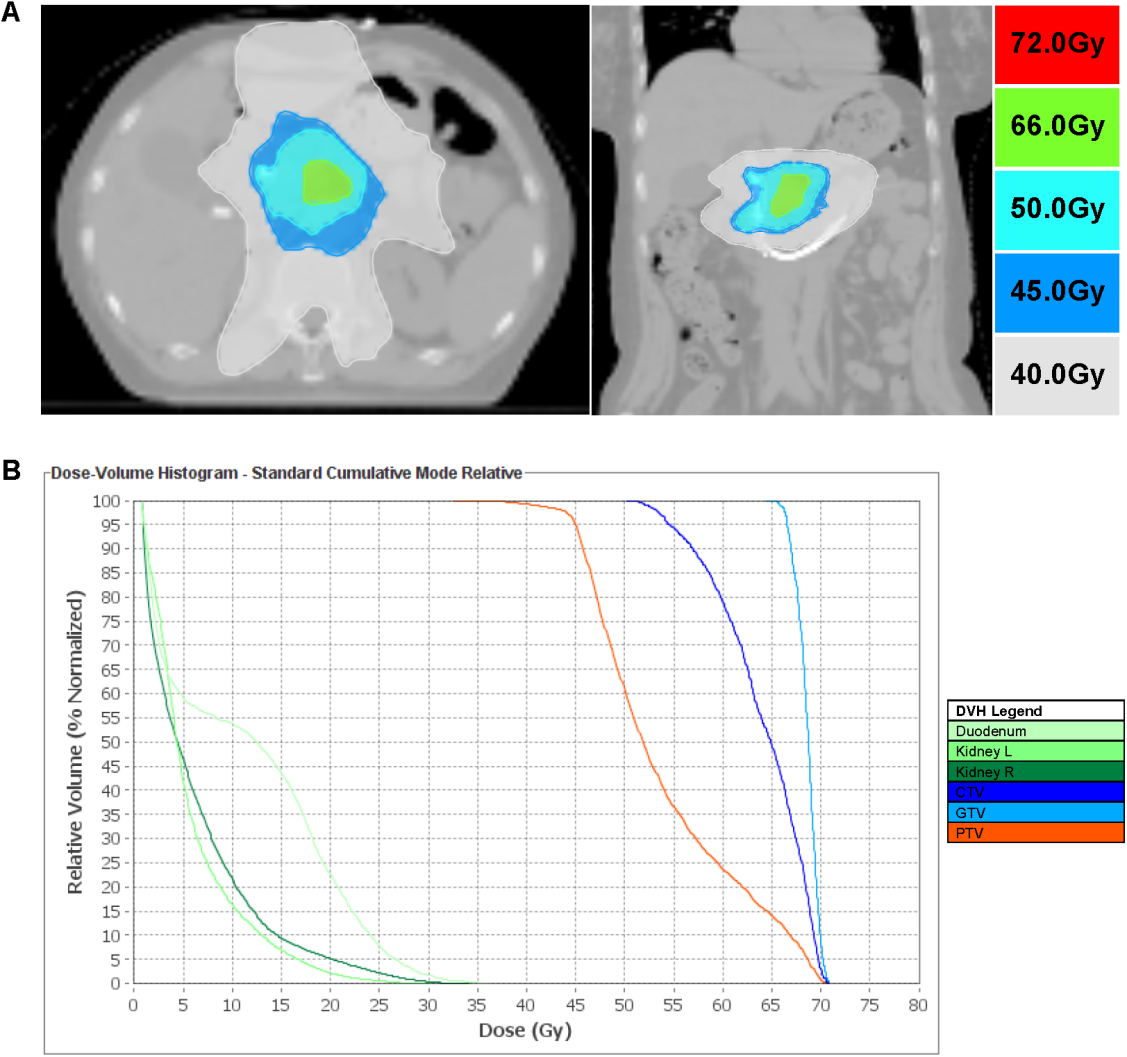
TOMO Radiotherapy Planning and Dose Distribution. **(A)** Dose escalation: GTV 66 Gy to pancreatic uncinate mass; CTV 50 Gy (GTV + 1 cm margin); PTV 45 Gy (0.5 cm respiratory margin). **(B)** Dose-volume histogram showing coverage for GTV, CTV, and PTV.

**Figure 4:**
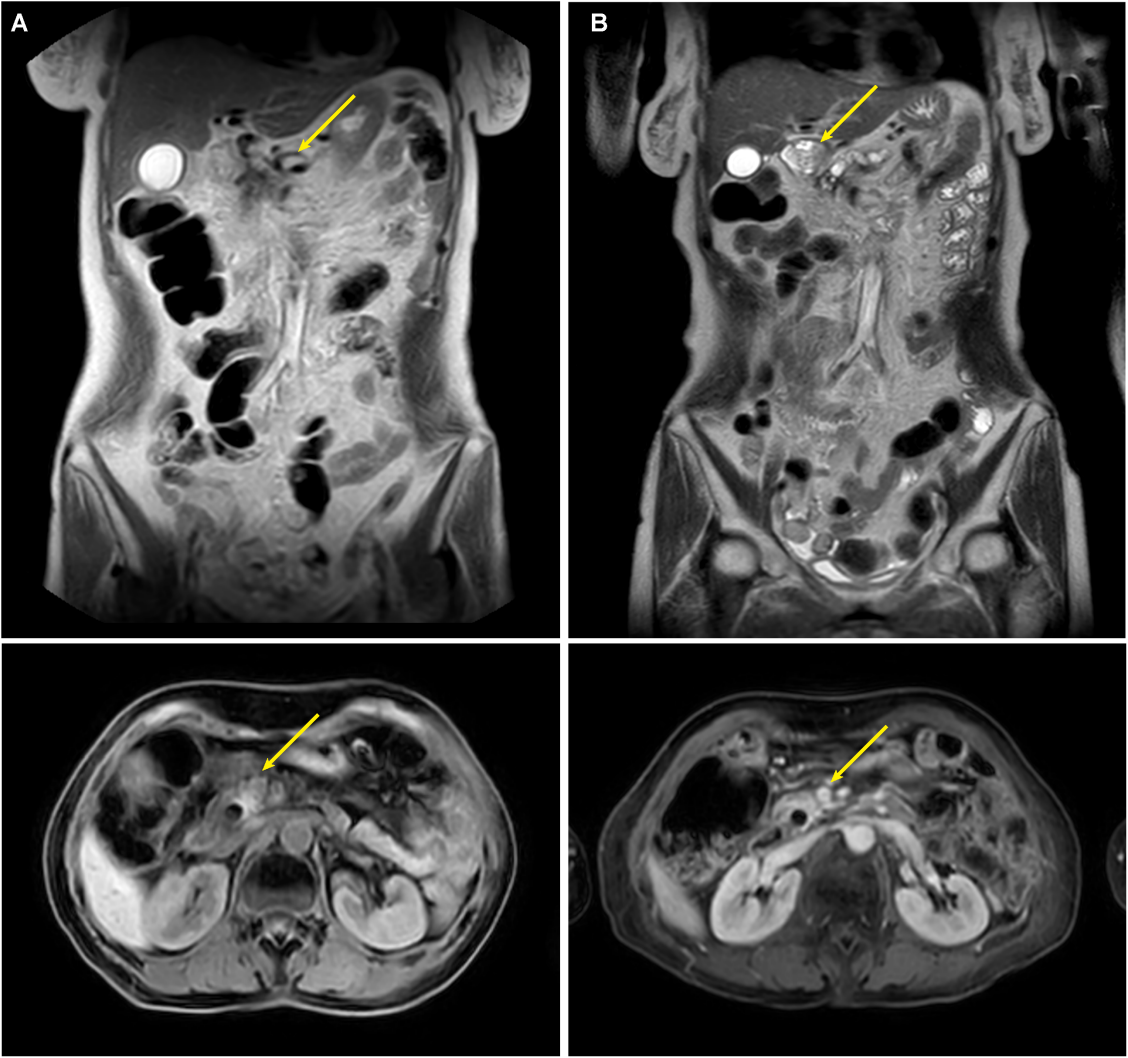
Follow-up MRI at 6 and 12 Months Post-PTCD. MRI demonstrates patent metal stent, with reduction of the pancreatic uncinate mass at 1 year **(B)** compared to 6 months **(A)**.

After jaundice resolution, we adopted a strategy prioritizing obstruction relief, local tumor control, and systemic therapy (12–14). The patient initially received the AG regimen (gemcitabine + nab-paclitaxel). However, recurrent hyperbilirubinemia led to intolerance, and multidisciplinary team (MDT) discussion prompted a switch to S1 (tegafur, 20 mg/day), nimotuzumab (weekly), and pembrolizumab (every three weeks). PTCD and seed stent placement rapidly alleviated jaundice, followed by TOMO delivering 66 Gy to the gross tumor volume (GTV), 50 Gy to the clinical target volume (CTV), and 45 Gy to the planning target volume (PTV) over 25 fractions, targeting the pancreatic primary lesion and regional lymph nodes (15) (**Figure 3**). Concurrently, the patient received weekly EGFR inhibitor nimotuzumab, PD-1 inhibitor pembrolizumab every three weeks, and long-term tegio (20 mg/day) (16). At one-year follow-up, the stent remained patent, MRI showed a 31% reduction in the target lesion (from 35 mm to 24 mm, **Figure 4**), and CA19–9 dropped to 272.9 U/mL (**Figure 2B**). The patient continues to be monitored. At one-year follow-up, TBIL was 18.2 μmol/L, DBIL was 9.8 μmol/L, CA19–9 decreased by 78%, and MRI showed tumor reduction (**Figure 4**). The patient reported a good quality of life (Karnofsky score: 90), with near-normal daily activities and minimal treatment-related discomfort.

## Discussion

MOJ severely compromises liver function due to biliary obstruction, with a median survival of less than three months (1). Although ERCP is the preferred initial approach (17), its imaging limitations for high-level obstructions and the short patency of plastic stents (3–4 months) often lead to failure (13, 18, 19). The bilirubin rebound in this case after 20 days was due to occlusion of the initial 8.5 Fr plastic stent, as confirmed by cholangiography showing no bile flow to the duodenum (**Figure 1A**). Replacement with a metal stent and iodine-125 seed strand resolved the obstruction, highlighting the superiority of this approach for sustained biliary decompression PTCD combined with metal stents offers sustained radial support, extending patency to 6–8 months (17, 20), while iodine-125 seeds further inhibit tumor infiltration, achieving patency of 10–12 months (8).

In this case, we employed a modified seed suspension technique, using retrievable biodegradable sutures to secure iodine-125 seed strands, which were removed after 6–8 weeks to minimize cumulative radiation risks and preserve space for subsequent radiotherapy. This approach reduced costs by 67% by eliminating the need for specialized radioactive stents or protective equipment, making it feasible for resource-constrained settings. Studies suggest that only 20–30% of primary hospitals in developing regions can perform permanent seed stent implantation (21). Our technique, relying on standard materials, is practical for wider adoption.

After biliary decompression, TOMO precisely targeted the pancreatic primary lesion, complemented by a personalized regimen of S1 (tegafur), nimotuzumab, and pembrolizumab achieving a 78% reduction in CA19–9 and survival beyond one year. This combination formed a synergistic antitumor network of radiosensitization, targeted therapy, and immune activation, reducing CA19–9 by 78% and extending survival beyond one year. Notably, prompt relief of biliary obstruction likely reshaped the local immune microenvironment by reducing immunosuppressive cell recruitment (e.g., myeloid-derived suppressor cells) through restored bile acid metabolism (1). Sequential seed and TOMO irradiation induced immunogenic cell death, releasing tumor antigens and activating systemic immunity (2). This effect synergized with tegafur’s cell cycle inhibition, collectively slowing tumor progression. Emerging evidence suggests PD-1 inhibitors enhance radiotherapy’s abscopal effect by upregulating tumor antigen presentation and CD8+ T-cell infiltration (3, 22). However, the absence of PD-L1 expression and tumor mutational burden (TMB) data limited our ability to predict immunotherapy response. High PD-L1 expression (>50%) and TMB (>10 mutations/Mb) are associated with better immunotherapy outcomes, seen in only 20–30% of pancreatic cancer patients (4, 19, 23). Limited tissue samples highlight the need for routine biomarker testing.

Patient Perspective: Per CARE guidelines, the patient’s perspective was assessed at one-year follow-up. The patient reported high satisfaction with the treatment regimen, citing minimal adverse effects and a Karnofsky score of 90, indicating near-normal daily function and excellent quality of life. She could perform routine activities, such as household tasks and social interactions, with minimal limitations, reflecting the clinical success of our multidisciplinary approach. This aligns with studies showing that effective biliary decompression and tumor control improve patient-reported outcomes (6).

Future multicenter randomized controlled trials are essential to validate the efficacy of PTCD with seed suspension and targeted-immunotherapy, particularly exploring personalized regimens based on PD-L1 and TMB profiling. Long-term follow-up should monitor stent displacement (incidence ~2.8%), seed migration, and risks of radiation-induced cholangitis. Artificial intelligence-assisted imaging could further enhance the precision of seed implantation and radiotherapy (24).

## Conclusion

Sequential PTCD and biliary seed stent placement, combining mechanical and radiotherapeutic effects, significantly prolongs stent patency and facilitates targeted immunotherapy, offering a novel approach for patients with ERCP failure or high-level obstructions. This case, with survival exceeding one year, underscores the clinical value of a multidisciplinary, stepwise strategy.

## Data Availability

The original contributions presented in the study are included in the article/supplementary material. Further inquiries can be directed to the corresponding authors.
